# Baseline comparison of three health utility measures and the feeling thermometer among participants in the action to control cardiovascular risk in diabetes trial

**DOI:** 10.1186/1475-2840-11-35

**Published:** 2012-07-11

**Authors:** Dennis W Raisch, Patricia Feeney, David C Goff, KM Venkat Narayan, Patrick J O’Connor, Ping Zhang, Don G Hire, Mark D Sullivan

**Affiliations:** 1 University of New Mexico Health Sciences Center, 1 University of New Mexico, Albuquerque, NM, 87131, USA; 2 VA Cooperative Studies Program, Clinical Research Pharmacy Coordinating Center, Albuquerque, NM, USA; 3 Wake Forest University Health Sciences, Medical Center Blvd, Winston Salem, NC, 27157-1063, USA; 4 Public Health Sciences and Internal Medicine, Wake Forest University Health Sciences, Medical Center Blvd, Winston Salem, NC, 27157-1063, USA; 5 Hubert Prof, Global Hlth & Epidemiology, Hubert Department of Global Health, School of Public Health, Emory University, Rollins School of Public Health, Grace Crum Rollins Building, 1518 Clifton Road, Atlanta, 30322, Georgia; 6 Senior Clinical Investigator, HealthPartners Research Foundation, Minneapolis, MN, USA; 7 Division of Diabetes Translation, National Center for Chronic Disease Prevention and Health Promotion, Centers for Disease Control and Prevention, Atlanta, GA, 30341, USA; 8 Wake Forest University Health Sciences, Medical Center Blvd, Winston Salem, NC, 27157-1063, USA; 9 Psychiatry and Behavioral Sciences, University of Washington, Box 356560, Seattle, WA, 98195, USA

**Keywords:** Diabetes mellitus, Type 2/*complications /physiopathology/psychology, Health status indicators, Randomized controlled clinical trial, Humans, Regression analysis, Glycemic control

## Abstract

**Background:**

Health utility (HU) measures are used as overall measures of quality of life and to determine quality adjusted life years (QALYs) in economic analyses. We compared baseline values of three HUs including Short Form 6 Dimensions (SF-6D), and Health Utilities Index, Mark II and Mark III (HUI2 and HUI3) and the feeling thermometer (FT) among type 2 diabetes participants in the Action to Control Cardiovascular Risk in Diabetes (ACCORD) trial. We assessed relationships between HU and FT values and patient demographics and clinical variables.

**Methods:**

ACCORD was a randomized clinical trial to test if intensive controls of glucose, blood pressure and lipids can reduce the risk of major cardiovascular disease (CVD) events in type 2 diabetes patients with high risk of CVD. The health-related quality of life (HRQOL) sub-study includes 2,053 randomly selected participants. Interclass correlations (ICCs) and agreement between measures by quartile were used to evaluate relationships between HU’s and the FT. Multivariable regression models specified relationships between patient variables and each HU and the FT.

**Results:**

The ICCs were 0.245 for FT/SF-6D, 0.313 for HUI3/SF-6D, 0.437 for HUI2/SF-6D, 0.338 for FT/HUI2, 0.337 for FT/HUI3 and 0.751 for HUI2/HUI3 (*P* < 0.001 for all). Common classification by quartile was found for the majority (62%) of values between HUI2 and HUI3, which was significantly (*P* < 0.001) higher than between other HUs and the FT: SF-6D/HUI3 = 40.8%, SF-6D/HUI2 = 40.9%, FT/HUI3 = 35.0%, FT/HUI2 = 34.9%, and FT/SF-6D = 31.9%. Common classification was higher between SF-6D/HUI2 and SF-6D/HUI3 (*P* < 0.001) than between FT/SF-6D, FT/HUI2, and FT/HUI3. The mean difference in HU values per patient ranged from −0.024 ± 0.225 for SF-6D/ HUI3 to −0.124 ± 0.133 for SF-6D/HUI2. Regression models were significant; clinical and demographic variables explained 6.1% (SF-6D) to 7.7% (HUI3) of the variance in HUs.

**Conclusions:**

The agreements between the different HUs were poor except for the two HUI measures; therefore HU values derived different measures may not be comparable. The FT had low agreement with HUs. The relationships between HUs and demographic and clinical measures demonstrate how severity of diabetes and other clinical and demographic factors are associated with HUs and FT measures.

**Trial registration:**

ClinicalTrials.gov Identifier: NCT00000620

## Background

Health utilities (HUs) are summary measures of health-related quality of life (HRQOL) for health states [1,2]. The HU scale ranges from 0.00 (dead) to 1.00 (perfect or optimal health) although some instruments allow for negative states, considered worse than death [1,3,4]. HUs are often captured indirectly from multiattribute HRQOL surveys in which responses are converted through scoring algorithms that are derived from direct measures of HUs. Examples of indirect HU instruments include the Health Utilities Index, Mark3 (HUI3) and the Health Utilities Index, Mark2 (HUI2) [3], developed using direct HU measures from the standard gamble and visual analog scale (VAS) techniques and the EuroQOL 5-dimensions (EQ-5D) [4], developed using direct HU measures from time trade-off and VAS techniques. The Short Form 6-dimensions (SF-6D) is derived from the Short Form 36 (SF-36), a generic measure of HRQOL, has also been converted into an HU based upon standard gamble techniques. The SF-6D expands the application of SF-36 as an indirect measure of HU [5].

The feeling thermometer (FT) is a visual analog scale (VAS) from 0 to 100 that is sometimes used as a direct measure of HU [6]‐[8]. The FT has a role in determining HUs, but with limitations, such as end-aversion bias (unwillingness of respondents to select the lowest health state) and FT results are ordinal values [9]‐[12]. Advantages of the FT as a measure of health status are ease of administration and simplicity, but its values require conversion, based upon classical direct HU measures [12,13].

Previously, researchers have identified differences in HUs derived from the SF-6D with those of the EQ-5D, VAS, or the HUI [14,15]. For example, SF-6D values have been shown to be higher than HUI values [14,16] in some studies and lower in another [17]. Since HUs are used to calculate quality adjusted life years (QALYs), these differences may be important in cost effectiveness analyses (CEA) of diabetes treatments as well as other conditions [18,19]. Disparate CEA results may be associated with method of calculating HUs, and its impact on QALYs [20]. Pickard et al. calculated HUs from 2 published studies using 10 different methods, based upon SF-36 and SF-12 data [20]. Based on an incremental cost difference of $2000 between treatments, the incremental CEA ratios ranged from $30,769 to $63,492 per QALY for an asthma study and $27,972 to $72,727 per QALY for a stroke treatment study [20]. Thus, the CEA decision could be dependent upon which method was used to calculate HUs. Previous research has shown that CEA results are sensitive to HU values. For example in a CEA modeling study of a diabetes prevention program, decreasing the improvement in HU values by 0.04 among participants, significantly increased the cost per QALY ratio by up to $10,000 [21].

Patient characteristics and disease severity should be associated with HUs. If they are not, the HU technique may be insensitive to important differences in the disease’s severity. Thus, the assessment of relationships between HUs and disease severity measures helps establish the sensitivity of a particular HU measure. Relationships between diabetes severity and HUI values have been found, with greater levels of complications associated with lower HUs [22]‐[24]. The SF-6D and VAS have been shown to discriminate severity of diabetes [25] and obesity [26,27]. Similar findings were found in a study among patients with coronary artery disease, which indicated that SF-6D and the HUI discriminate groups defined by gender and symptoms, as well as responsiveness to changes in angina pain over time [14].

Our first objective was to compare HUs calculated using standardized scoring algorithms, the HUI2, HUI3 and the SF-6D and the FT values among participants enrolled in the Action to Control Cardiovascular Risk in Diabetes (ACCORD) trial. Our second objective was to assess relationships between each HU and demographic characteristics, clinical measures, diabetic complications, and comorbidities.

## Patients and methods

ACCORD was designed to compare the effect of intensive versus less intensive control of glucose, blood pressure and lipids on CVD event rates among participants with type 2 diabetes who are at risk for cardiovascular events. This randomized, controlled clinical trial was conducted over 8 years (estimated mean patient follow-up of 5.6 years) among 10,251 participants at 77 study sites in the United States and Canada (http://www.accordtrial.org)[28]. The intensive glucose control arm of the study was discontinued in February 2008 due to increased mortality compared to less intensive control and the blood pressure and lipid control arms were completed in 2009 [29].

CEA was incorporated into ACCORD as a sub-study [30]. All sites obtained institutional review board approval for ACCORD and the CEA sub-study. Participants were randomly selected for participation in the CEA sub-study and completed informed consent. There were 4311 participants enrolled in the CEA sub-study and, nested within this sample, 2053 participants completed HRQOL instruments at baseline and 12, 36, and 48 months. For this cross-sectional study we examined baseline results from the SF-36, Version 2 (RAND Corporation, Santa Monica, CA), the Health Utilities Index (Health Utilities Inc., Dundas, Ontario, Canada), and the FT. ACCORD inclusion/exclusion criteria are described elsewhere [31]. All HRQOL sub-study participants who completed baseline measures of all three instruments were included, but forms with missing data were excluded from the analyses.

### Analyses

The SF-36 was converted into the SF-6D HU score, using techniques previously described [32]. The HUI was scored according to both the HUI2 and the HUI3 algorithms, resulting in two HU scores [3]. The FT represents how the patient feels on a 0 to 100 scale. We converted the FT values to 0.00 to 1.00 (dividing by 100) because values range from below 0.00 to 1.00 for the other instruments in the study, Demographics, physiologic and laboratory measures, complications, and comorbidities were obtained from baseline case report forms. All data have been edited according to data capture and verification procedures established for the ACCORD trial. For all statistical analyses, a two-sided alpha was set at 0.05.

For our first objective, we calculated pair-wise intra-class correlation coefficients (ICCs) using one-way random models to determine level of agreement between HU instrument scores as well as FT values [33]. We also divided the scores into quartiles, and determined whether values from each HU or the FT, fell within the same quartiles. We analyzed the level of agreement between the pairs of instruments using chi square tests. We also calculated the mean differences per patient (and 95% confidence intervals) between pairs of instruments to determine the extent of variation between the HUs.

For the second objective, we developed multiple regression models with HUs or FT values as dependent variables and demographics, clinical measures, and diabetes complications as independent variables. Categorical variables were entered into the model as indicator variables (for example, presence of characteristics = 1, absence = 0). We used a stepwise, forward selection process with *p* < 0.5 and report the variables that were significant contributors to the model at *p* < 0.05 All data computation and analyses were performed using SAS Software, V9.1 (SAS Institute). We tested normality of the distributions of HUs and FT using Kolmogorov-Smirnov tests and assessed Goodness of Fit for the models based on the significant F values.

## Results

After removing surveys with missing responses, the final sample sizes included in the study were n = 1951 (95.0%) for SF-6D and n = 2035 (99.1%) for HUI2, HUI3, and FT. For analyses comparing correlation and agreement among HU measures, we included only observations with all four measures. For regression analyses, we used any observation with that particular HU or FT score, regardless of the completeness of the other HU scores.

Table 1 displays the alignment of demographic variables for this sub-study sample and the overall ACCORD trial. There are no significant differences. The mean ± standard deviation (median) were: SF-6D = 0.684 ± 0.085 (0.696), HUI2 = 0.806 ± 0.156 (0.849), HUI3 = 0.707 ± 0.258 (0.778), and FT = 0.748 ± 0.168 (0.78). The values were normally distributed (Kolmogorov-Smirnov tests).

**Table 1:** Demographic and physiologic variables of all ACCORD participants versus health-related quality of life (HRQOL) sample

**Baseline Characteristic**	**Overall** **ACCORDn = 10,251**	**HRQOL Samplen = 2053**
Mean Age, Yrs	62.2 ±6.8	62.2 ±6.7
Median Age, Yrs	62	62
% Female	38.6	39.6
% White	64.8	65.9
% Black	19.3	19.4
% Hispanic	7.2	6.8
% Minority	37.6	36.3
Highest Level of Education	.	.
% Less than High School	14.8	13.9
% High School Graduate	26.4	26
% Some College	32.8	33.2
% College Graduate or More	26	26.9
Cigarette Smoker	.	.
% Never	41.8	41.2
% Former	44.2	45.6
% Current	14	13.3
% Secondary Cardio vascular Disease Status	35.2	36.1
Mean Duration of Diabetes, Yrs	10.8 ± 7.8	11.1 ± 7.8
Median Duration of Diabetes, Yrs	10	10
Mean glycated hemoglobin (HbA1c), %	8.3 ± 1.1	8.3 ±1.1
Median HbA1c, %	8.1	8.1
Mean Fasting Plasma Glucose, mg/%	175.3 ±56.2	177.1 ±57.6
Mean Serum Creatinine, mg/ml	0.9 ±0.2	0.9 ±0.2
Mean Glomerular Filtration Rate, ml/min	91.1 ±27.1	91.7 ±31.3
Mean Weight, lbs	206.2 ±41.1	207.5 ±41.7
Body Mass Index, kg/m^2^	32.2 ±5.5	32.4 ±5.5
Waist Circumference, inches	42 ±5.5	42.1 ±5.5
Mean Systolic Blood Pressure (mm/HG)	136.4 ±17.1	136.2 ±17.1
Mean Diastolic Blood Pressure (mm/HG)	74.9 ±10.7	74.5 ±10.9
% On Any Hypertension Medications	85.4	85.5
% On Any Angiotensin-receptor converting enzyme Inhibitors	53	52
% on Beta Blockers	29.3	30.3
Mean Low Density Lipoprotein (LDL) (mg/dl)	104.9 ±33.9	104.3 ±34.0
High Density Lipoprotein (HDL) (mg/dl)	41.9 ±11.6	42.1 ±11.7
HDL among Females (mg/dl)	47 ±12.6	47.3 ±12.6
HDL among Males (mg/dl)	38.6 ±9.6	38.7 ±9.7
Total Cholesterol (mg/dl)	183.3 ±41.9	182.8 ±41.3
Non-HDL Cholesterol (mg/dl)	141.4 ±41.4	140.7 ±40.9
% with High Triglyceride	23.3	23.3
Median Triglycerides (mg/dl)	155	156
% On Statins	62.1	63.5

**Figure 1:**
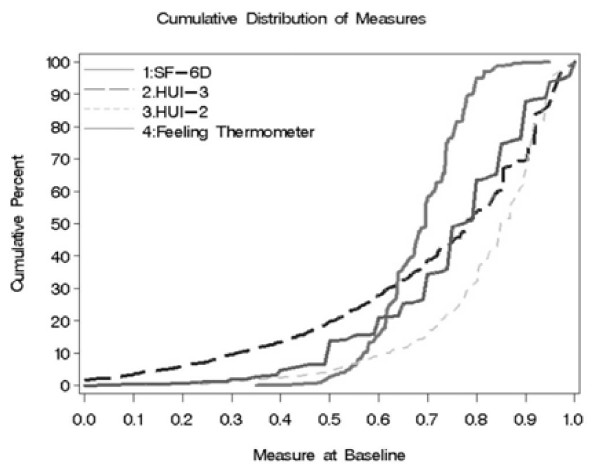
Health utility and feeling thermometer scores by measurement technique, with cumulative distributions.

Figure 1 displays the cumulative distribution plots of all HU instruments and the FT. The SF-6D scores encompassed a narrower range than the other instruments. The middle 2 quartiles (26% to 75%) for SF-6D range from 0.62 and 0.74, versus 0.65 to 0.85 for FT, 0.76 to 0.92 for HUI2, and 0.57 to 0.92 for HUI3. This is demonstrated by the steepness of the slope of the middle of the distribution curve for the SF-6D (Figure 1). Another difference is that participants’ responses to the FT tended toward specific values listed on the scale, increments of 0.05 and 0.10. This is shown by the stepped appearance of the distribution plot for the FT. We note that 1044 (51.3%) of FT scores fell on a multiple of 0.10, and another 714 (35.1%) fell on other multiples of 0.05, for a combined total of 1758 (86.4%). Thus, the FT scale differentiated most scores by 0.05 point intervals, whereas calculated scores from other instruments can be any possible value within the scoring range.

ICCs between instruments represent strong agreement between HUI2 and HUI3 (0.733, 95% confidence interval (CI) 0.732-0.734). However there was poor agreement between FT and SF-6D (0.245 CI: 0.241-0248). There was only fair agreement between HUI3 and SF-6D (0.313 CI: 0.266-0.358), HUI2 and SF-6D (0.437 CI: 0.436-0.438), FT and HUI2 (0.337 CI: 0.332-0.342), and FT and HUI3 (0.337 CI: 0.317-0.353). All ICCs were statistically significant (*p* < 0.01).

**Figure 2:**
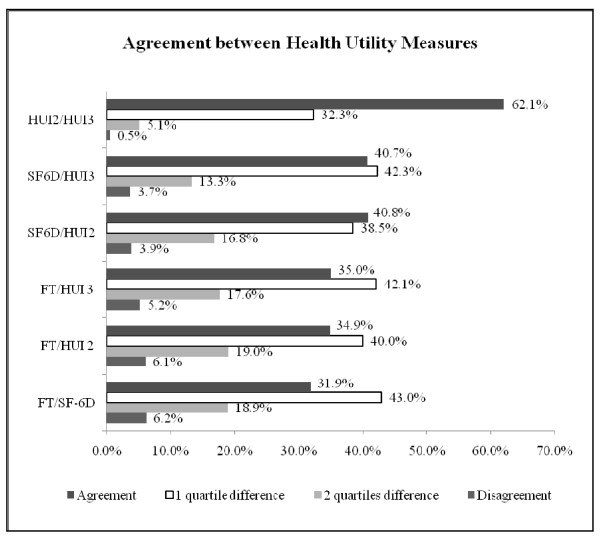
**Within quartile agreement between instruments by quartile.** HUI = Health Utilities Index, FT = Feeling Thermometer, SF-6D = Short Form-6 Dimensions, Disagreement = top quartile of one score, lowest quartile of other score, Significantly higher % agreement: HUI2/HUI3 versus all others (*P* < 0.001):, SF-6D/HUI3 versus FT/HUI3, FT/HUI2, FT/SF-6D (all *P* < 0.001), SF-6D/HUI2 versus FT/HUI3 (*P* = 0.008), FT/HUI2 (*P* = 0.003), FT/SF-6D (*P* < 0.001), FT/HUI3 versus FT/SF-6D (*P* = 0.04).

Figure 2 displays comparisons between each pair of instruments by quartile. The greatest disparities between instruments, with participant scores in highest quartile of one instrument but the lowest quartile of another, were between the FT versus all other instruments. Similarly more of the FT scores were 1 or 2 quartiles different from HU values. The significant differences in percent agreement between pairs of HU instruments and the FT are listed. HUI2 and HUI3 were in agreement significantly more than all other pairs. The SF-6D/HUI3 and SF-6D/HUI2 were in agreement significantly more than the any of the FT pairs. The mean differences (95% CIs) were: SF-6D/HUI2 = −0.124 (−0.386 to 0.137). SF-6D/HUI3 = −0.024 (−0.466 to 0.417), SF-6D/FT = −0.065 (−0.386 to 0.256), FT/HUI2 = −0.058 (−0.424 to 0.308), FT/HUI3 =0.041 (−0.450 to 0.533), and HUI2/HUI3 = 0.10 (−0.205 to 0.404). Similar to the disagreements between quartiles and ICCs, the confidence intervals reflect large differences between scores for some patients.

The results of the adjusted multivariable regression models for each instrument are shown in Tables 2 and 3. The models were statistically significant (*p* < 0.001) and explained 6.1% to 7.8% of the variances. The variables common to all models were female gender, secondary CVD, diabetes duration, and current smoking, which were associated with significantly lower values. Education, total cholesterol, and low-density lipoprotein were associated with significantly higher values in all models except the FT. It is interesting that greater age was associated with slightly higher values for all models except HUI3. It could be that older patients had lower values of other predictors that were significant negatively and that the age variable adjusted for those differences. We note that for each model where age was a predictor, it was entered at a higher step (data available upon request). Other researchers have found positive relationships between age and HUs [34]. Aspirin consumption was associated with significantly higher values for HUI2 and FT. Hispanic race was associated significantly lower values for SF-6D and HUI3. African American race was associated with significantly higher FT scores. Increased body mass index (HUI2 and HUI3) or waist circumference (FT and SF-6D) was associated with significantly lower values.

**Table 2:** Multivariable regression results by instrument *

**Variable**	**SF-6D**		**Health Utilities Index 3 Score**		**Health Utilities Index 2 Score**		**Feeling Thermometer**	
	**Slope**	***P*- value**	**Slope**	***P*- value**	**Slope**	***P*- value**	**Slope**	***P*- value**
	**Estimate**		**Estimate**		**Estimate**		**Estimate**	
0-Intercept	0.756	**<.0001**	1.038	**<.0001**	0.907	**<.0001**	1.012	**<.0001**
Aspirin	.	**.**	.	**.**	0.014	**0.041**	0.015	**0.041**
Body mass index	.	**.**	−0.006	**<.0001**	−0.004	**<.0001**	−0.002	**0.062**
NonWhite	.	**.**	.	**.**	.	**.**	−0.039	**0.000**
Waist circumference (inches)	−0.002	**<.0001**	.	**.**	.	**.**	−0.003	**0.010**
Age	0.001	**0.043**	.	**.**	0.001	**0.007**	0.002	**0.001**
Black	.	**.**	.	**.**	.	**.**	0.060	**<.0001**
Total cholesterol	0.000	**0.001**	−0.001	**<.0001**	−0.001	**0.000**	.	**.**
Duration of diabetes (years)	−0.001	**0.002**	−0.002	**0.005**	−0.001	**0.001**	−0.001	**0.011**
Educational level	0.005	**0.006**	0.023	**<.0001**	0.014	**<.0001**	.	**.**
Female	−0.024	**<.0001**	−0.040	**0.001**	−0.030	**<.0001**	−0.043	**<.0001**
Glycated hemoglobin	.	**.**	.	**.**	.	**.**	−0.014	**<.0001**
Low density lipoprotein	0.000	**0.008**	0.001	**0.001**	0.000	**0.017**	.	**.**
Secondary cardiovascular disease	−0.013	**0.001**	−0.069	**<.0001**	−0.032	**<.0001**	−0.018	**0.021**
Smoking	−0.009	**0.003**	−0.040	**<.0001**	−0.027	**<.0001**	−0.019	**0.001**
Hispanic	−0.027	**0.001**	−0.067	**0.004**	.	**.**	.	**.**
Variance explained by model	**6.1%** **(*P*** **< 0.001)**		**7.7%** **(*P*** **< 0.001)**		**7.6%** **(*P*** **< 0.001)**		**7.7%** **(*P*** **< 0.001)**	

**Table 3:** Values for Significant Categorical Variables in Multivariable Regression

**Variable**		**SF-6D**	**HUI3 Score**	**HUI2 Score**	**Feeling Thermometer**
Aspirin	No	0.660	0.648	0.774	0.750
	Yes	0.667	0.667	0.788	0.765
NonWhite	No	0.666	0.678	0.789	0.784
	Yes	0.661	0.636	0.773	0.731
Black	No	0.664	0.634	0.779	0.720
	Yes	0.663	0.681	0.783	0.796
Education	Less than High School	0.655	0.616	0.759	0.757
	High School Graduate	0.662	0.662	0.781	0.757
	Some College	0.663	0.641	0.776	0.755
	College Graduate	0.673	0.709	0.809	0.763
Female	No	0.673	0.677	0.793	0.776
	Yes	0.654	0.637	0.769	0.740
Secondary cardiovascular disease	No	0.671	0.689	0.795	0.766
	Yes	0.656	0.625	0.767	0.750
Smoking	No	0.672	0.692	0.805	0.776
	Previous	0.669	0.686	0.795	0.768
	Current	0.650	0.594	0.743	0.729
Hispanic	No	0.675	0.668	0.782	0.743
	Yes	0.652	0.646	0.780	0.773

## Discussion

There were significant differences between the 3 methods of measuring HUs and the FT.

### HU comparisons with previous research

We note that each sample of patients are dissimilar and thus are not directly comparable to our results, however we did identify similarities to previous studies. The range of mean HU values across the instruments (0.684 to 0.818) in our study were similar to those reported in Action in Diabetes and Vascular Disease study (ADVANCE) which used the SF-6D and the EQ5D (0.678 to 0.801) [35]. The mean FT value in our study (0.748) is similar to the mean value reported among participants of United Kingdom Progressive Diabetes Study (0.74) [36] and among patients with obesity (0.751) [27]. However, our mean FT value was higher than the value reported in the Cost of Diabetes in Europe -Type 2 (CODE-2) (0.628, converted from 62.8 to have similar decimal places to our results) [37] and the FT value found by Matza, et al. among patients with diabetes (0.623) [38]. The differences may reflect study instrument administration techniques as well as differences in study samples. In a study designed to determine the impact of hypothetical diabetes medication outcomes, the patients’ FT scores from diabetes were lower than ACCORD participants, who had higher rates of hypertension (85.4% vs. 37.2%), while body mass index (BMI) was similar (32.2 vs. 31.3) [38]. A study using time-tradeoff measures found a mean HU of 0.76 for conventional glucose control [18], which is similar the FT HU obtained from ACCORD participants at baseline.

### Measurement characteristics: comparisons between instruments

The cumulative distributions (Figure 1) help elucidate differences between the methods. The FT scores were concentrated at the interval values listed on the instrument, for example, multiples of 0.05 or 0.10. This finding may suggest lower sensitivity to changes in HRQOL smaller than 0.05, which is smaller than what has been suggested as a clinically important difference in HU (0.03) [39]. The limitations of the FT when compared to HU measures have been previously described [13,40].

HUs obtained from the SF-6D varied by the smallest range among the middle 50% of the patients (from quartiles >25% to <76%), the difference was only 0.12 points for the SF-6D versus 0.21 for FT, 0.18 for HUI2, and 0.35 for HUI3. The finding suggests that the scoring algorithm for the SF-6D may be less sensitive to differences in HUs among participants whose scores are within this range. A narrower range of scores for the SF-6D has been previously documented when compared to the EQ-5D, and is considered a potential limitation of the SF-6D [41,42]. The narrow scoring range of the SF-6D was also demonstrated among rheumatoid arthritis participants when compared with HUI3 and EQ-5D [43] and among participants in an implantable defibrillator study when compared with the HUI3 [17]. The HUI2 scoring range was also narrow among the middle 50% of patients (Figure 1), as was previously shown among rheumatoid arthritis patients [43]. The HUI3 cumulative distribution plot is the most gradual across the mid-range scores, suggesting more differentiation between participants within the middle quartiles.

We note several differences between the HUI 2 and HUI3. The HUI3 includes scales for vision, hearing, and speech versus a sensation domain for the HUI2; has separate domains for dexterity and ambulation versus mobility for HUI2; and uses different questions for emotion and pain. Therefore the domain scores are not directly comparable. Only the domain of cognition uses the same questions, but cognition is scored differently between the 2 instruments (6 levels in HUI3 compared to 4 levels in HUI2) Furthermore, the domain scores are then entered into different scoring algorithms for the HUI2 and HUI3, resulting in different values. We note that since the HUI3 differentiates patients more broadly within the middle quartiles, it may be a better method for scoring the HUI in the ACCORD population. The ACCORD CEA sub-study planning committee selected the HUI3 a priori [30].

The ICCs found between HU and FT values, with the exception of the expected higher value between the HUI2 and HUI3 scoring algorithms, provide a summary statistic showing poor or fair agreement between the instruments. Fair agreement between SF-6D and HUI3 was found among patients in an implantable cardiac defibrillator trial (ICC = 0.45) [17] and a percutaneous coronary intervention trial (ICC = 0.40) [14]. Our results by quartile describe these discrepancies more specifically. Comparing the FT and HUI2, 6.1% (n = 123) participants would be measured as being in the highest quartile by one instrument, while scoring in the lowest quartile of the other (Figure 2). Furthermore, an additional 19.2% (n = 383) of comparisons between the FT and the HUI2 differ by two quartiles.

The mean (± standard deviations) differences per participant showed similar results, with average differences from 0.100 (HUI2/HUI3) to −0.122 (SF-6D/HUI2) and 95% confidence intervals as great as −0465 to 0.417 (SF-6D/HUI2). These large discrepancies indicate that the choice of HU instrument could impact results of the overall CEA. Specifically, one instrument might show increased HU over time while another shows a negative or no impact in the same participant. Such discrepancies between HUs have been identified previously among a primary care population in East Asia [44] and among rheumatoid arthritis patients in British Columbia [43]. A longitudinal analysis is needed to determine the full impact of these discrepancies in regard to sensitivity to changes in physiologic diabetes measurements (e.g. glycated hemoglobin or cardiovascular complications).

### Relationships between HUs and clinical and demographic variables

In multivariable analyses, we identified significant relationships between HUs and FT values. Comorbidities negatively associated with HUs were presence of CVD, current smoking, and obesity measured by BMI or waist circumference. Either waist circumference or BMI were significant for all HU instruments and both were significant for FT. When BMI was significant it may have addressed the variance in HUs associated with waist circumference and vice versa. Previously, in a study of the impact of long-term diabetic complications on HRQOL, BMI was a significant predictor in all regression analyses of SF-36 domains with the exception of mental health [45]. Similarly, in CODE-2, obesity was a significant predictor of VAS scores [37]. Relationships between VAS and obesity have also been shown among patients with obesity without a diagnosis of diabetes [46]. History of CVD was significantly associated with lower HU and FT values. Significant relationships between HU and CVD among patients with diabetes have been shown in other studies [35]‐[37,45].

Among physiologic measures, total cholesterol and low-density lipoprotein were significantly associated with lower values for all HU instruments, but not for the FT. Glycated hemoglobin was only associated with FT values. None of the renal function measures (serum creatinine, micro- and macro-albuminuria) were significant in any models. Regarding use of blood pressure, lipid, and glycemic medications; none were significant in multivariable models. Our models were similar to a study of diabetes-related complications, which used the EQ-5D in 1143 Canadian participants [34]. Specifically, the researchers reported significant relationships between HU and duration of diabetes (negative), age and male gender (both positive), and CVD complications (myocardial infarction and stroke, negative).

Recently a simulation study was conducted to demonstrate the impact of complications on life expectancy among patients with Type 2 diabetes using data from the Fenofibrate Intervention and Event Lowering in Diabetes (FIELD) study [47]. The simulation study showed that an HU less than 1.00 at baseline was associated with increased all-cause mortality and lower quality adjusted life expectancy [47]. Occurrences of diabetic complications were associated with a mean decrease of 0.045 HU (95% CI = −0.073 to −0.017), as measured by the EQ-5D [47]. The greatest impact on HU was stroke (−0.165 HU. 95% CI = −0.246 to −0.0840). Similarly, in our multivariable models we found secondary CVD to be associated with significantly lower HU at baseline (Tables 2 and 3). The association varied by type of measure, SF- 6D (−0.015), HUI3 (−0.064), and HUI2 (−0.028).

We note that clinical and demographic factors associated with HU are similar to results of an observational trial of predictors of hypertension management [48]; in which persons with diabetes, obesity and Hispanic ethnicity were found to have decreased blood pressure control. The study found the lowest percent of patients with controlled blood pressure control (23%) among diabetic persons with obesity [48].

Adequate goal attainment of CVD risk factors continues to be illusive among persons with Type 2 diabetes. In a study of the data from National Health and Nutritional Examination Survey from 1999 to 2008, goal attainment improved significantly for low density lipoprotein (LDL), from 29.7% to 54.4%, but control of hypertension did not significantly improve (47.6% to 55.1%; *P* = 0.1333) even though significantly more patients were receiving antihypertensive medications (35.4% to 58.9%; *P* < 0.0001) [49]. Prevalence of hypertension was not significantly increased from 1999 to 2008 (66.6% to 74.2%; *P* = 0.3724) [49].

Another concern is under-diagnosis of diabetes among CVD patients. Researchers reviewed health records of all Danish myocardial infarction (MI) patients who were not previously diagnosed with diabetes to identify the initiation of glucose lowering medications within 1 year after discharge [50]. The rates increased from 19.6 to 27.6 per 1000 person year from 1997 to 2001, at which time the rates leveled off through 2005 [50]. These rates were much lower than expected, since other researchers had shown higher rates of abnormal glucose tolerance among MI patients. However a recent population study of screening for diabetes and CVD found no difference in HU among screened versus non-screened populations [51].

A limitation of the study is that ACCORD participants are a select group (i.e. mean age > 62, type II diabetics who met study inclusion criteria and were at risk for CVD); thus, our results are not generalizable to all other individuals with type 2 diabetes. Further research would be needed to make comparisons to other patient groups with type 2 diabetes. This analysis is limited to baseline measures only; our results do not indicate how values may be influenced by changes in diabetes severity or the study interventions over time.

We identified significant differences in HU values obtained from the SF6D, HUI2 and HUI3. Since differences in HU values could impact CEA results, the type of patient preference measure used is an important consideration in designing and interpreting CEAs. Although we found statistically significant relationships between HUs and demographic and clinical variables, the variances explained by the models were relatively small (6.1% to 7.7%).

## Abbreviations

ADVANCE: Action in Diabetes and Vascular Disease study; ACCORD: Action to Control Cardiovascular Risk in Diabetes; BMI: Body mass index; CVD: Cardiovascular disease; CI: Confidence interval; CEA: Cost effectiveness analyses; CODE-2: Cost of Diabetes in Europe -Type 2; EQ-5D: EuroQol-5 Dimensions; FT: Feeling thermometer; FIELD: Fenofibrate Intervention and Event Lowering in Diabetes; HUI2 and HUI3: Health Utilities Index, Mark II and Mark III; HU: Health utility; HRQOL: Health-related quality of life; ICCs: Intra-class correlation coefficients; LDL: Low density lipoprotein; MI: Myocardial infarction; QALYs: Quality adjusted life years; Short Form: Short Form; SF-6D: Short Form-6 Dimensions; VAS: Visual analog scales.

## Competing interests

The authors declare that they have no competing interests.

## Authors’ contribution

DR drafted and revised the manuscript. PF and DH performed the statistical analyses. DR conceived of the study. MS, DG, PZ, KN, and PO participated in its design, reviewed and provided input for drafts of the manuscript. All authors read and approved the final manuscript.
